# Laser-Induced Fluorescence for Monitoring Environmental Contamination and Stress in the Moss *Thuidium plicatile*

**DOI:** 10.3390/plants12173124

**Published:** 2023-08-30

**Authors:** Kelly Truax, Henrietta Dulai, Anupam Misra, Wendy Kuhne, Peter Fuleky, Celia Smith, Milton Garces

**Affiliations:** 1 Department of Earth Sciences, University of Hawai‘i at Mānoa, Honolulu, HI 96822, USA; hdulaiov@hawaii.edu (H.D.); anupam@hawaii.edu (A.M.); milton@isla.hawaii.edu (M.G.); 2 Savannah River National Laboratory, Aiken, SC 29831, USA; wendy.kuhne@srnl.doe.gov; 3 UHERO and the Department of Economics, University of Hawai‘i at Mānoa, Honolulu, HI 96822, USA; fuleky@hawaii.edu; 4 School of Life Science, University of Hawai‘i at Mānoa, Honolulu, HI 96822, USA; celia@hawaii.edu

**Keywords:** bryophyte, metal contamination, environmental stress, metal detection, laser-induced fluorescence, image processing

## Abstract

The ability to detect, measure, and locate the source of contaminants, especially heavy metals and radionuclides, is of ongoing interest. A common tool for contaminant identification and bioremediation is vegetation that can accumulate and indicate recent and historic pollution. However, large-scale sampling can be costly and labor-intensive. Hence, non-invasive in-situ techniques such as laser-induced fluorescence (LIF) are becoming useful and effective ways to observe the health of plants through the excitation of organic molecules, e.g., chlorophyll. The technique presented utilizes images collected of LIF in moss to identify different metals and environmental stressors. Analysis through image processing of LIF response was key to identifying Cu, Zn, Pb, and a mixture of the metals at nmol/cm^2^ levels. Specifically, the RGB values from each image were used to create density histograms of each color channel’s relative pixel abundance at each decimal code value. These histograms were then used to compare color shifts linked to the successful identification of contaminated moss samples. Photoperiod and extraneous environmental stressors had minimal impact on the histogram color shift compared to metals and presented with a response that differentiated them from metal contamination.

## 1. Introduction

Metal distribution in the environment from anthropogenic sources has long been a concern, but regulations within the last few decades have made monitoring of industry, mining, and urban development more important. Heavy metals in high concentrations have been shown to bind with soil and organic matter long after their release and deposition, making monitoring more recent contaminant accumulation more challenging [1,2]. Focusing on the atmospheric deposition of these metals of interest can help to distinguish current events in the environment from historic ones [1,3,4]. Observation of biota has been used since the 1960s for monitoring metals through bioaccumulation, with flora specifically serving as habitat monitors and pathways for bioremediation [5]. Among these biomonitors are mosses, which have traditionally aided in the monitoring of the atmospheric deposition of heavy metals due to their lack of a true root system, hence no accumulation of historic contaminants from underlying soils [6,7].

Mosses are a resilient group with a simple cellular structure and are found in a wide variety of biomes [8,9,10]. Though mosses are good accumulators of environmental contaminants, uptake of a specific metal varies with moss size and lipid structure of the cellular wall [11]. Micronutrients like Cu, Zn, and Ni are typically preferentially taken up and incorporated into cellular structures due to their use in metabolic processes, while non-essential elements like Pb and Hg may be adsorbed but generally end up trapped as particulate matter on the surface, bound to chelating sites, or deposited around cells [12]. Non-essential metals are usually toxic to plants in any amount, but high levels of micro-nutrients have been shown to also alter chloroplasts and total chlorophyll content [5,8,13,14]. High levels of Cu were consistently found across studies to cause changes in chlorophyll content and affect the ratio of chl -a and -b [15]. These changes in chlorophyll have been documented in mosses and other plants using spectrophotometry [16,17,18,19], Pulse-Amplitude-Modulation (PAM) [20,21,22] Schreiber, 2004; Brooks & Niyogi, 2011; Haidekker et al., 2022, and laser-induced fluorescence (LIF) [23].

Though the literature [14,15] denotes chlorophyll changes due to mental stress, shifts in chlorophyll content can also occur in response to environmental stressors. Al-Radady et al. showed that physical stress can affect the efficiency of plant tissues to retain elements [24]. Documentation of chlorophyll fluorescence has helped detect stress conditions in plants [25,26,27] and the addition of nitrogen fertilization in agriculture [28]. Experiments have also detected shifts in red fluorescence (chlorophyll) due to severe drought [29] and the effects of water stress [30]. Each of these parameters is likely to affect plants during in situ analysis, making it necessary for any monitoring method to distinguish between metal-induced stress and environmental effects from water stress, drought, and nutrient introduction. Another inherent factor affecting plants is the length of the photoperiod [31], which is different across geographic locations and times of year. This paper documents how metal-induced stress is distinguishable from environmental variance.

Laser-induced fluorescence (LIF) is an emerging technique applied in the biological sciences to monitor shifts in photosynthetic physiology and chlorophyll-a in plants, leaves, and algae [32,33]. In this work, fluorescence is induced via a pulsed laser at a rate of nanoseconds to capture the short lifetime fluorescence of organic material. The laser excites molecules using electromagnetic radiation, which is absorbed and quickly emitted as a spontaneous emission of light [34,35]. LIF offers the ability for in-situ non-destructive measurements without the need for close-up devices, such as PAM, or reliance on long-distance satellite information that comes with their shortcomings [36,37]. LIF could replace traditional sampling and allow larger-scale, repeated measurements of the same habitat. The benefits of LIF when compared to near-IR (infrared) spectroscopy are that it is a non-destructive method, utilizes portable instruments, and can take measurements under daylight and at considerable distances [30,38,39,40].

Previous work [41] strove to develop a method of identifying Cu contamination in moss using image analysis of LIF response. The effort employed the “Standoff Biofinder” [42], which was a pulsed Nd:YGa dual laser system (green 532 nm laser and UV 355 nm laser) fired at a nanosecond rate. It was found that differentiation of Cu concentrations at varying μmol/cm^2^ levels was possible based on the comparison of color histograms of treated moss samples to those of control samples. However, that proof-of-concept work was limited to testing only one metal, Cu, at concentrations 2–3 magnitudes above common environmental levels of concern. Therefore, while it proved the feasibility of applying LIF to metal detection in biota, improved sensitivity would be required to reach detection at environmentally relevant nmol/cm^2^ levels.

For this study, adjustments were made to the design and functionality of the laser system to prevent power loss, improve reflectivity in the laser lens, and block unwanted wavelengths with the addition of a short pass filter to the upgraded CMOS camera. These adjustments improved the LIF detection limits of Cu to the nmol/cm^2^ level, well within environmental detection levels of interest. This improved system was renamed “CoCoBi”, for Color Compact Biofinder [43] and has been documented to detect 1 ppm of chlorophyll in ethanol from a single laser pulse excitation. The unit can be used effectively up to a 3 m distance with a field view of 60 cm. The system can also distinguish between biofluorescence and mineral luminescence using time-gated measurements.

One goal of this research was to demonstrate the applicability of the CoCoBi at environmentally relevant (nmol/cm^2^) Cu levels. Efforts expanded to apply the methodology to other metals and to observe if multiple environmental stressors, such as drought and length of photoperiod, could affect the moss response and image analysis. Cu was used for its known response, but Zn and Pb were also included because they are known to have been released from industrial, mining, and highway sources [44,45]. Another reason for examining these heavy metals is their frequent detection in trace metal assessments conducted for environmental and public health [46].

## 2. Materials and Methods

### 2.1. Laboratory Procedure

The moss *Thuidium plicatile* Mitt. is a moss species endemic to Hawai’i [47] (Appendix A) and was collected along the Wa’ahila Ridge Trail and State Recreational Area (21.307°, −157.797°) on 15 February 2021. The forested area represents an uncontaminated environment along the Southeastern part of the Koʻolau mountain range beneath the Honolulu Watershed Forest Reserve. The samples were washed and cleaned of forest litter before being placed on trays, which were moved within a laboratory grow tent for incubation. Moss samples throughout the time were kept at 18–20 °C, 50–60% relative humidity, and 14–17 W/m^2^ ambient light (1400–1800 lux), with a default daylight length of 10 h using a VIPARSPECTRA P1000 LED full spectrum, dimmable grow light. 10 h was selected as an average daytime to simulate average conditions to allow the methodology to be generally applicable regardless of geographic location.

Three metals of interest (Cu, Zn, and Pb) were administered individually and as a mixture of the three metals for comparison. Three environmental stressors of interest were selected and included drought, overwatering (drowning), and high nutrient regime. The effects of long (14 h), short (6 h), and dark (0 h) photoperiods were also tested. With the inclusion of control, the preparation of samples was conducted two weeks before experiments, leading to the separation of moss onto eleven different plastic trays. Each tray contained moss covering an area of 316 cm^2^ which was then placed in the growth tent. A two-week acclimation period before the start of the experiment provided ample time for any moss physiological response to laboratory conditions and transplantation to trays to stabilize. We adapted previously published methodology [41] to determine when moss was beyond the point of acclimation shock from the transfer to new growth conditions. Specifically, moss health was monitored via stabilization of water uptake to 30 mL per tray per day and through measurement and reaching of consistent humidity levels between 50–60%. These two parameters must stay constant for one week to confirm that equilibrium in moss growth has been reached within the growth tent [41].

During the experimentation period, each tray of moss was removed from the tent for about 0.5 h for treatment and imaging, after which it was immediately returned. On non-treatment days, all moss (save the drought sample) were given 30 mL of deionized water (DI). DI was added on non-treatment days to maintain a constant watering regime, ensuring that any moss response recorded resulted from the experimental addition of metals, nutrients, or a stress response. The experiment was seven days in total, including a control day and treatment with metal three times at increasing toxicity levels with 48 h between treatments. For the three increasing doses, Cu, Zn, and Pb were administered at 1 nmol/cm^2^, 10 nmol/cm^2^, and 100 nmol/cm^2^ every 48 h, respectively. Compounds of CuCl_2_, ZnCl_2_, and Pb(NO_3_)_2_ were used as chloride and nitrate forms of the metals that have not been shown to affect metal uptake in mosses [48]. The trial was treated with a mixture of metals combined at each of the three doses. Wetting of the samples always occurred within 5 min before imaging.

The nutrified sample was given a single dose of nutrients on the first day of the experiment (Miracle-Gro AeroGarden Liquid Plant Food 4-3-6; Appendix B). No other moss samples were given nutrients of any kind during the acclimation period and during experimentation to ensure that any reaction we document could only be attributed to the stressors tested. The drought sample ceased to receive water on the first day of the experiment to watch the effect of withholding water for a week. The overwatered sample received 30 mL of DI each morning before imaging and again before imaging 12 hrs later, totaling twice as much watering as all other samples. Photoperiods for all samples were kept at 10 h except for the long photoperiod (14 h), the short photoperiod (6 h), and the dark photoperiod (no light) treatments. Each of the photoperiod samples was given one week under its new light conditions before its first imaging. Table 1 outlines the treatments for each sample over the seven days of the experiment. Due to space constraints and the time needed for imaging, a maximum of 4 trials was run per week. This broke the samples into three groups: those treated with metals, changing environmental conditions, and finally, photoperiod conditions. The control trial was imaged during the same experimental period as the “environmental condition” group.

### 2.2. LIF Imaging Using the CoCoBi

Each group of moss samples was imaged starting on day 1 to collect a baseline control for each tray. Moss receiving metal solutions were dosed on days 2, 4, and 6 before imaging. The nutrified sample received excess nutrients on day 1, while all other environmental group samples maintained the same treatment throughout the seven days of the experiment. The moss trays were always imaged after wet deposition when applicable, and imaging was conducted every 12 h. In the case of the overwatered sample, wetting occurred before all imaging sessions. Imaging was conducted in 30-min windows in order of trial number, and those imaging times were held consistent for each sample between 6 and 8 a.m. and 6 and 8 p.m. The standard 10 h period of daylight within the growth tent began at 8 a.m. and ended at 6 p.m. This allowed imaging to occur during the final hours of the nighttime (6–8 a.m.) and after a full daytime (6–8 p.m.).

Further details of the laser system are described in Misra et al. [43], but in short, the CoCoBi is a dual laser system that uses both green and UV lasers. Images were collected with the Baumer Camera Explorer software, which allows the CMOS camera to be synchronized with the 112 ns pulses of the Nd:YAg laser. Baumer Camera Explorer allowed for the adjustment of the camera’s exposure, gain, and time delay [42]. Due to the slight angle of the CoCoBi’s alignment when imaging, five shots were taken from the front of the moss tray (each corner and the center), and then the tray was rotated 180° so the “back” of the moss could be imaged, producing ten images in total (five front and five back) every 12 h. Approximately each image captures a moss area of 5 cm by 7.6 cm.

### 2.3. Data Analysis

#### 2.3.1. Single-Color Comparison

**Figure 1:**
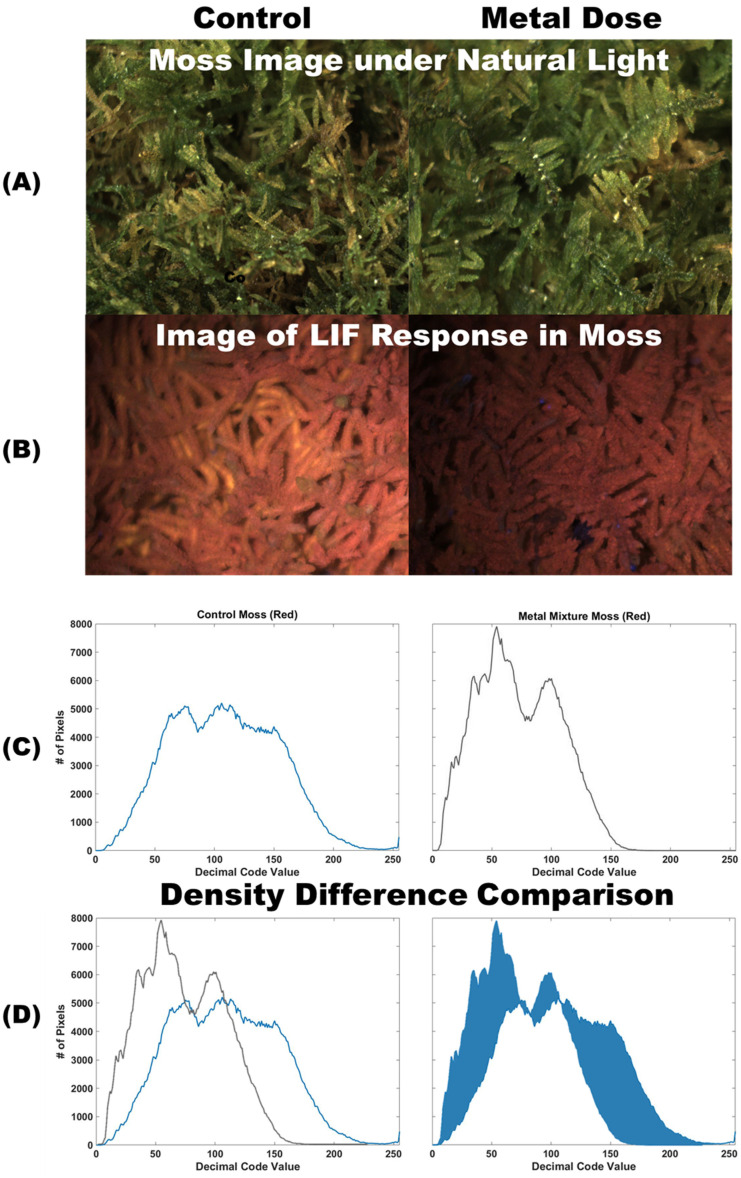
(**A**) Images of moss samples showing a control sample and a sample dosed with Cu under natural light. (**B**) Shows images of the same samples exposed to LIF with the control sample on the left and a contaminated sample on the right. (**C**) Color histograms of the red color channel are calculated from the LIF images of moss by extracting the decimal code value for every pixel with the control shown in blue and the metal dosed shown in black. (**D**) The two histograms can be compared by overlapping the two curves (**left**) and calculating the areas of difference (**right**) to find the density difference. Alternatively, the images can be compared using the DTW method to find the minimal direction of change needed in the x/y direction to fit the contaminated histogram to the control.

Once the images of LIF response in each moss sample were collected (Figure 1B), RGB (Red, Green, and Blue) pixels within each image were extracted to create density histograms of the relative abundance of each decimal code value from 0 to 255 for each color channel (Figure 1C). The decimal code abundances were normalized using the total pixel count to create a percent abundance curve. Once calculated, the profiles of these histograms were then used to assess the difference between curves (density difference; Figure 1D).

(1) $$Difference=1-\left(\sum minimum\left|trial\left(x\right),\;control\left(y\right)\right|\right)$$

where x represents the color intensities for the corresponding trial, and y represents the same for the control images, with the sum of the absolute minimum between the two curves being found for each decimal code value (x axis). An alternative method was also used to dynamically time warp one curve to fit another (DTW) [49].

(2) $$D\left(i,j\right)=\left|x\left(i\right)-y\left(j\right)\right|+min\left\{\begin{array}{c} D\left(i+1,j\right)\\ D\left(i+1,j+1\right)\\ D\left(i,j+1\right) \end{array}\right.$$

where x and y represent strings of data and *i* and *j* represent the length of each string so that *D*(*i*,*j*) equals the best alignment distance between all data points along the lengths of *x* and *y* [49]. Sample images of moss from the metal group trays before treatments and all control sample images from the week of experimentation were included for comparison. This experiment captured ten images per tray every 12 h of the experiment and had a total of 193 control images sampled. Due to the 3-dimenional nature of the imaging, it is imperative that the same pixel area is used when comparing images to ensure the removal of any artifacts or errors associated with the laser set-up. For example, in Figure 1B there are brighter and darker sections of the image moving from the bottom right corner to the top left corner of both images. The change in gradient is the same between images and is due to the proximity of the camera to the laser. As long as the pixel area being compared is the same *x* and *y* coordinates for both images, this artifact is automatically correct for the image analysis.

The developed workflow relies upon batch processing of the individual images stored in organized folders. Functions created in Matlab (2021a) access the data stored in these folders and process them to extract RGB color histograms into a table for batch processing to compare all ten images of a single-day treated trial to all 193 images of the control to find all possible iterations of difference. From these comparisons, a trial mean, standard deviation of the sample images, and standard error of the trial mean can be calculated for each day for each trial and stored in arrays for further use. The mean and standard deviation for imaging sessions (every 12 h) were used to compare all trials to the control using a Welch’s *t*-test to check if two populations of images are similar enough that we cannot reject that they are the same [50]. The *t*-test was used for each color channel for each trial compared to the control based on the day of the trial.

#### 2.3.2. Multi-Color Comparison

One drawback of using the density difference method to find differences between images is that it can only be used to compare single-color histograms. Truax et al. [41] found that DTW could be used for both single- and two-color analysis, which improved the sensitivity of detection and separation of individual samples from each other and the control. Because of the use of the improved CoCoBi LIF system, DTW was used again for two-color analysis. Previous work had only explored the two-color combination of red and green color channels. To observe the possible reaction of moss to various stressors, all three two-color combinations were considered in this experiment (RvG, GvB, RvB).

However, even this approach does not allow for the use of all information from the three available color channels. Thus, to improve upon previous methods and to determine if the change amongst the three-color channels was due to a specific stressor, a new method was developed for testing. Single-color values extracted from the density difference method for each trial imaged on each day of the experiment and compared with its control were used to create color ratios. This calculated difference is represented in Equations (3)–(5) for each color variable as *R_D_*, *G_D_*, and *B_D_*, where *D* denotes the color histogram difference. Each color difference (*R_D_*, *G_D_*, and *B_D_*) was then divided by the sum of all three color differences, representing the total color difference between treated and control images.

(3) $$\frac{R_{D}}{\left(R_{D\;}+G_{D}+B_{D}\right)}$$

(4) $$\frac{G_{D}}{\left(R_{D\;}+G_{D}+B_{D}\right)}$$

(5) $$\frac{B_{D}}{\left(R_{D\;}+G_{D}+B_{D}\right)}$$

The resulting ratios represent the relative color change as a fraction of the total change. This was done for all trials, including the control. The mean of each color ratio was calculated to observe if a pattern of separation was discernable within the data. A Welch’s *t*-test [50] was used to quantitatively determine if any color ratio pattern for a given trial deviated from the control across the 7-day experiment. Deviation from the control is only recorded as true if it exceeds a confidence interval of 99%. Suppose the results of a contaminated sample of moss fall within the 99% confidence interval for the control. In that case, we can be confident that the contaminated sample falls within the control population represented in the confidence interval. If an image collected falls outside this 99% confidence interval for the control, we can say with high statistical certainty that it is not a control sample, and the stressor effect can be observed.

## 3. Results

The research was divided into three parts based on the methodology developed in previous work [23]. Part one focused on the laboratory treatment and care of the moss samples by acclimatizing them in a growth tent and performing metal dosing over seven days. Part two was the use of LIF and capturing color images of the moss response. Finally, the collected images were analyzed by extracting all pixels and creating histograms of each RGB (Red, Green, Blue) color channel. The following results utilized an intersection density difference method and dynamic time warping (DTW) by comparing images of each trial to the control to quantify the moss response for stressor identification.

### 3.1. Single Color Analysis Using Density Difference

**Figure 2:**
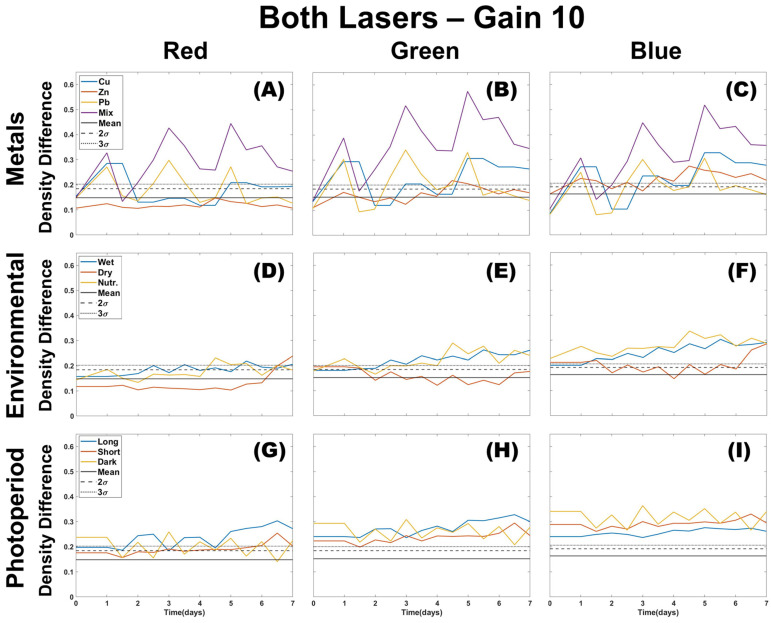
Single color density difference analysis for all color channels (R,G,B) of images taken using both lasers of the CoCoBi for metal (**A**–**C**), environmental (**D**–**F**), and photoperiod (**G**–**I**) trials with the control mean and confidence intervals included.

Experimental testing of three stress groups (metals contamination, environmental stress, and photoperiod length) using images collected from two lasers (green and UV) at a gain of 10 (Figure 2) reveals that red (R) and green (G) colors of images of mosses affected by environmental stressors show little deviation from the control sample. Long periods of overwatering or excess nutrients resulted in an RGB profile change, but their mean and variance show the results either fall within the control’s natural variation or slightly rise above the 99% confidence interval. When comparing the three-color channel results, R appears the least separated from the control for mosses treated with metals, with G having the greatest deviation. However, the G color channel also shows an increasing deviation from the control for over-wetting and treatment with excess nutrients, eventually crossing the control 3σ threshold. This separation from control is not present in the R color channel. The blue (B) color channel shows the same deviation for over-wetting, and nutrification shows a consistent separation from the control after the moss received a single dose of nutrients. Drought appears to have the least deviation from the control until the final day of the experiment, and prolonged testing would be needed to determine a response representative of in situ seasonal drought conditions.

The effects of photoperiod length seem to have a greater separation from the control than environmental stressors, with shorter photoperiod having the least deviation in R and G color channels (Figure 2 panels G–I). Long and dark photoperiods have similar deviations for R and G color channels, but a larger separation from the control is apparent in the B channel (Figure 2, panel I). The B color channel also appears most responsive to all metal treatment days during the experiment (days 1, 3, and 5; Figure 2, panels A–C). Photoperiod and metals were not tested together. Therefore, it is uncertain how their profiles would be if both occurred simultaneously.

**Figure 3:**
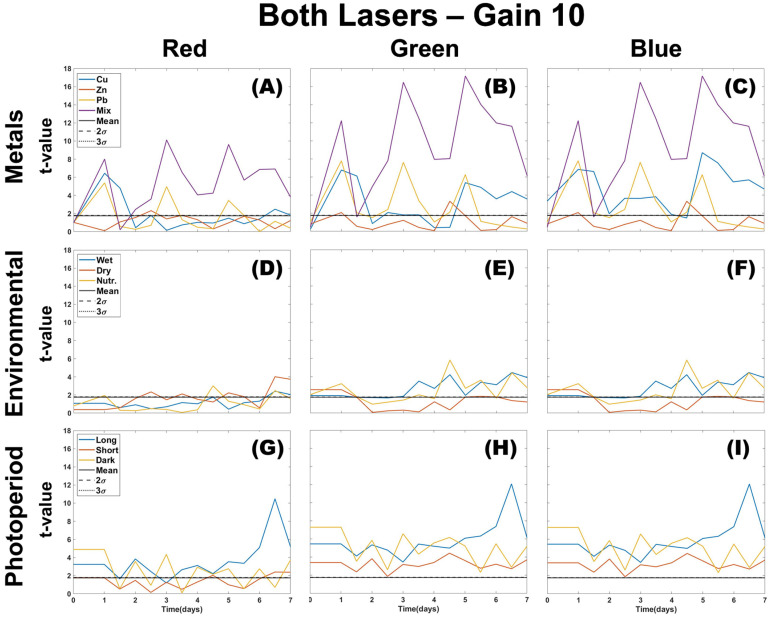
Welch *t*-test was applied to the results of single-color density difference values (Figure 2) for all color channels (R,G,B) of images taken using both lasers of the CoCoBi for metal (**A**–**C**), environmental (**D**–**F**), and photoperiod (**G**–**I**) trials.

The Welch *t*-test results in Figure 3 suggest that image processing using the blue color channel presents a statistically significant deviation from the control for all stressor types. Statistically significant differences between both the green and blue channels from the control can be found in Cu profiles for each treatment day and Zn on the first day of dosing. Lead shows separation in all color channels on all dosing days, whereas the mixture of metals shows separation every day after the first dose in all color channels. Overwetting, drought, and nutrients have lower “t” values when using the red color channel and are only separate from the control on some days in blue and green color channels. As observed visually in Figure 3, the *t*-test confirms that photoperiod length profiles could negatively interfere with metal identification by overlapping with a similar statistical difference compared to the control in all color channels when using the density difference method.

### 3.2. Two Color Analysis Using DTW

**Figure 4:**
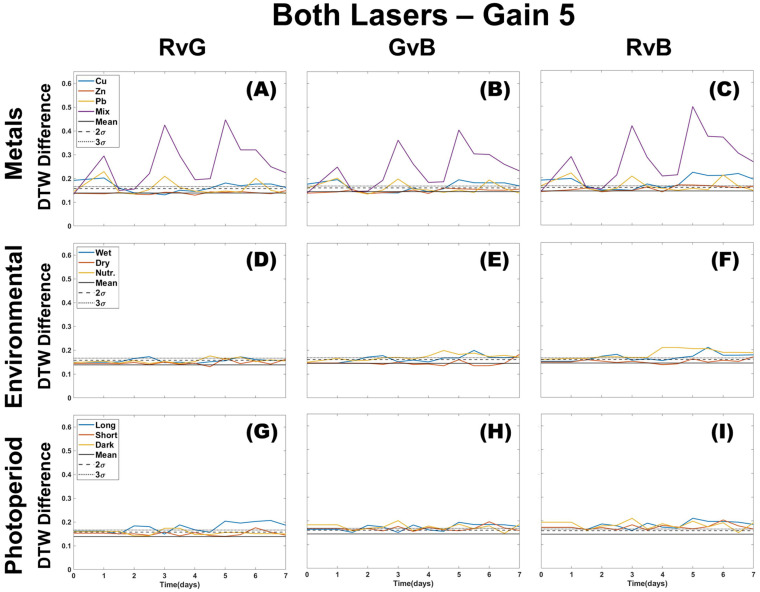
Two-color DTW for all color channels (RvG, GvB, RvB) of images taken using both lasers of the CoCoBi for metal (**A**–**C**), environmental (**D**–**F**), and photoperiod (**G**–**I**) trials with the control mean and confidence intervals included.

Figure 4 shows the profiles for images processed using the two-color DTW analysis using both lasers at a gain of 5. All environmental and photoperiod stressors are mostly bound within the natural variation of the control. The presence of metal is observable by statistical deviation in all color combinations (RvG, RvB, or GvB) except for Zn. Zn shows deviation only in GvB, but this overlaps with trends observed in environmental or photoperiod tests. Cu differs from controls in RvG and RvB, and the mixture of metals shows a deviation in all three colors. Environmental and photoperiod stressors almost always show only one two-color deviation. The shorter and dark photoperiods deviate in GvB, while the long photoperiod in RvG. DTW provides clear identification of metals without interference due to environmental or photoperiod effects. The mixture of metals is easy to observe and separate from the individual metal doses. There does appear to be an initial response on the first treatment day (day 1) for Pb, Cu, and the mixture of metals, but Cu is more distinguishable in GvB, whereas the Pb response stays constant throughout the color combinations. The most significant observation that can be made is that the environmental and photoperiod profiles do not show large variation and are only minimally separate from the control, if at all. Deviation is more likely in the RvB and GvB for environmental stressors, while photoperiod may be more pronounced, though slightly, in RvG and GvB. This could indicate that environmental stressors are more likely to be distinguishable through the blue color channel, while photoperiod can be identified using the green color channel. Metals can be identified regardless of color channel and will present with a similar profile, which is not observed with the other stressor types.

**Figure 5:**
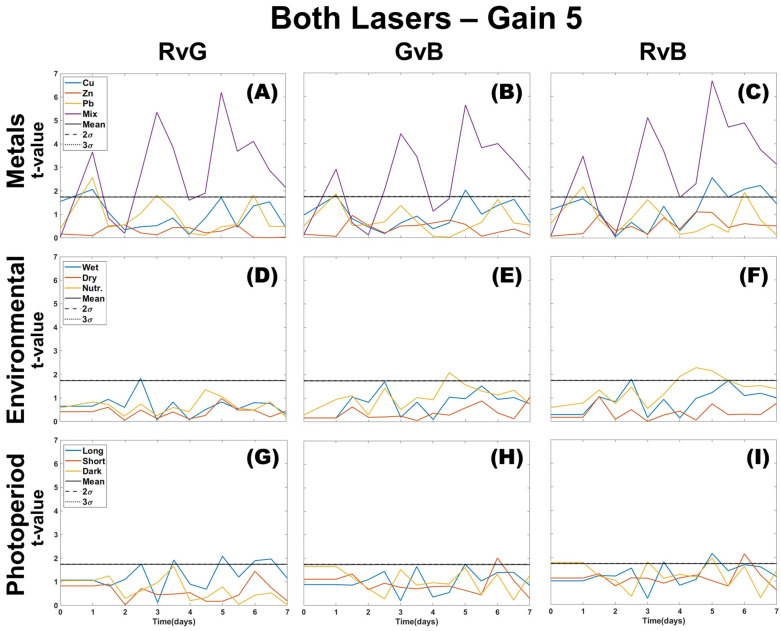
Welch *t*-test applied to results of two color DTW values for all color channels (RvG, GvB, RvB) of images taken using both lasers of the CoCoBi for metal (**A**–**C**), environmental (**D**–**F**), and photoperiod (**G**–**I**) trials.

The Welch’s *t*-test was also applied to the two color DTW values. As shown in Figure 5, the plotted profiles show that the use of DTW is more effective than density difference at minimizing the observed deviation of environmental or photoperiod stressors from the control. The nutrified samples appear to deviate in RvB and GvB, while the long photoperiod sample deviates in RvG and GvB. Again, we see the presence of deviation for environmental stressors when the blue color channel is present and in photoperiod treatments when the green color channel is present. This method is quite effective for delineating differences between a mixture of metals and individual metal profiles. However, it might be difficult to determine if a stressor was caused by nutrients or Cu, with Pb and Zn being the most difficult to identify. Therefore, this approach may be best applied when determining if there is a presence of multiple metals within a given sample.

### 3.3. Multi-Color Ratios as a Means of Stressor Determination

**Figure 6:**
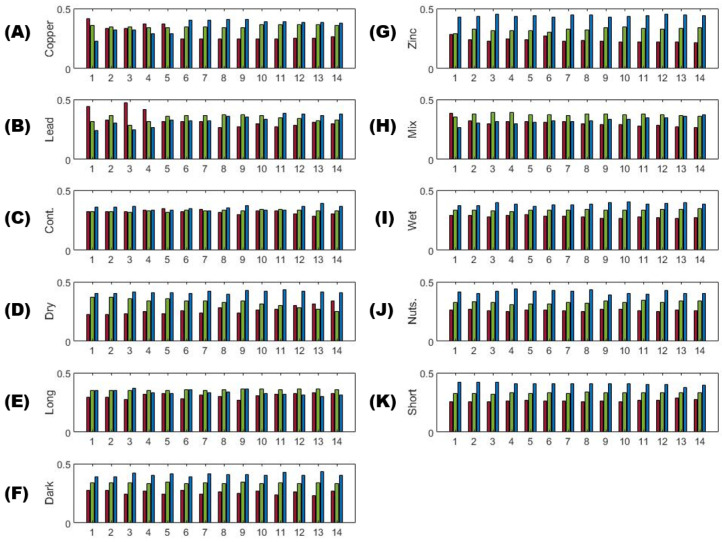
Use of color ratios from density difference analysis to compare the 11 trials every 12 h of the 7 day experiment starting with 1 and ending at 14 (2 imaging sessions a day). Values used are calculated by dividing individual color channels (R, G, B—color corresponds to bar plots) by the sum of their difference from the control. The trials are displayed as (**A**) Copper, (**B**) Lead, (**C**) Control, (**D**) Dry/Drought, (**E**) Long photoperiod, (**F**) Dark photoperiod, (**G**) Zinc, (**H**) Mixture of metals, (**I**) Wet/Over watering, (**J**) Nutrients, and (**K**) Short Photoperiod.

Multi-color analysis has the potential to capture the relationship between all color channels. It is hypothesized to be at least as valuable as a two-color analysis for individual metal differentiation. A contribution from each color to the overall image change compared to the control can be expressed as a fraction or percent of a single-color change within the sum of all color changes. We also derived this ratio for the control by using the ten images collected each day for the control sample taken during the 7-day experiment and comparing it to the total number of control images (193) as was done with the other trials. The color fraction relationship is shown as bar graphs in Figure 6, which shows the results for each trial from the density difference analysis of both lasers at a gain of 10 (2.1). The short and dark photoperiods, high nutrient regime, and over-wetting all show a similar pattern of increasing fraction of contribution from red (lowest) to green and finally to blue (highest). The long photoperiod and the control show even contributions from all three colors. The long photoperiod does appear to have a slightly larger contribution from green, while the control has a slightly larger contribution to the overall image change from the blue color. With time, the image changes for the drought treatment change from green to red dominated, while the blue color change contribution stays about the same. The metal profiles are the most distinct, with Zn showing the largest change contributed by blue color and decreasing red contribution. Pb and Cu start with red color change domination, which shifts over time to green and blue. The mixture of metals maintains a higher contribution from change in the green channel and is distinct from all other trials.

**Figure 7:**
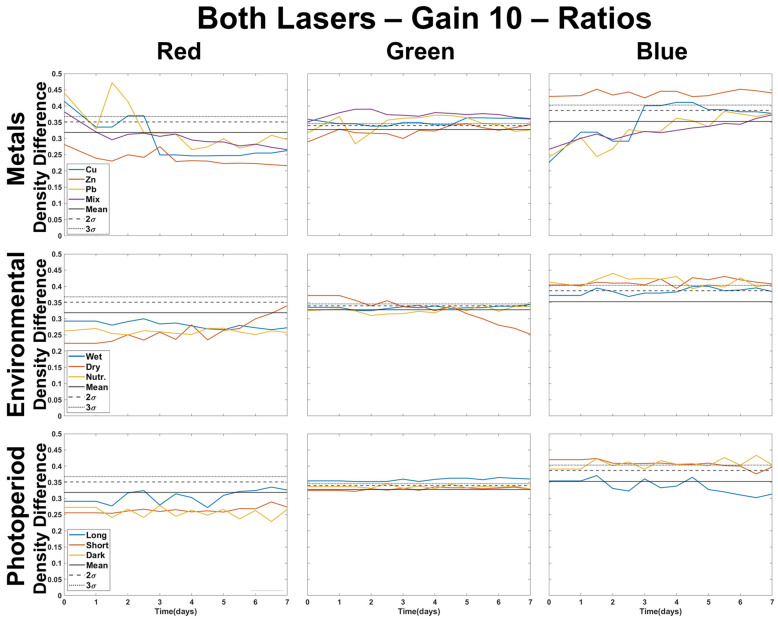
Ratios of density difference for individual color to the total difference.

The pattern of ratios (Figure 7) confirms that the highest fraction of green and blue channel contribution to overall change is associated with metal contamination of Cu, Pb, and the mix. Zn only deviates in the blue channel, which overlaps with all photoperiods and environmental stressors. Overwetting, however, is most similar to the control and the long photoperiod, which may interfere with metal identification. These results are more straightforward than the one-color analysis using the density difference method, but both methods show valuable information that, when combined, could make separation between stressor types easier.

## 4. Discussion

The presented experiments illustrate that with carefully tailored methodology, it is possible to use LIF to identify metal contamination in the moss *Thudium plicatile* Mitt. at environmental levels (nmol/cm^2^), even when compared to plants under environmental stress. The samples were kept within the same growth tent, with constant parameters for climate where possible. To ensure that any change observed was due to stressor type, a control was run for seven days alongside the environmental group. Each moss tray was imaged before exposure to its stressor type to characterize each sample. At this point, each sample has been in the grow tent for at least two weeks, and because no significant variation was observed, it is expected that our methods are not sensitive to baseline biological variability between moss trays. To further account for natural variability, their initial images before stress was induced were included within the total control image set. In the case of the same metal dosing being administered in replicate [41], we saw multiple small doses had the same impact as a single large dose. Therefore, we did increase increments of dosing (1, 10, 100 nmol/cm^2^) based off of those previous [41]. As for the applicability of individual R, G, and B colors, changes in the blue color channel appeared to be the most sensitive in providing separation from the control and individual dosing days when observing the metal group. Changes in the red channel were the least pronounced, but this could result from the natural variability within the fluorescence of chlorophyll response within the red spectra. Therefore, separation in green and blue channels proves to be very useful for identifying metal contamination.

Single color density difference analysis allows for the detection of multiple stressors. The parameters are not sensitive to environmental stressors, but the photoperiod in Figure 2 showed a major overlap with individual metals, which could create difficulty in identification. Metals and photoperiod were not tested on the same sample, so it is uncertain if they would have additive effects. *T*-test confirms that the photoperiod length could cause interference with any metal identification, but the metals themselves have a two or three-colour deviation from the control on dosing days. When considering color ratios, green channel separation from the control is associated with Cu and Pb metal contamination (Figure 7). Zn is difficult to discern as it overlaps with the elevated blue channel, which is more consistent with environmental or photoperiod stressors. Using a Welch *t*-test combined with color ratios makes it possible to distinguish the type of stressor present (metal, environmental, photoperiod) when using DTW with two-color analysis, but it lacks enough specificity to distinguish individual metals.

Two-color DTW results shown in Figure 4 have environmental and photoperiod profiles that rarely deviate from the natural variation of the control. However, plotting the *t*-test values for each two-color combination proved useful in separating a combination of metals from other stressor types (Figure 5). As with density difference, we can observe that photoperiod and environmental stressors mostly only deviate in one of the two-color combinations. Zn is still difficult to identify, though a RvG and GvB combined deviation may indicate Zn presence. More experiments would be needed for the validation of this approach. Cu shows early deviation from control in RvG and RvB at lower treatment levels, but at higher doses, Cu-treated samples deviate instead in the RvB and GvB. This is distinct from Pb and the mixture of metals, which deviate from the control in all color combinations. Because of their similar deviation, it is possible that identifying Pb from the mixture of metals would be difficult, and more experiments would be needed to confirm moss fluorescence response to other combinations of metals. However, based on the results, it is hypothesized that these mixtures of metals are identifiable from individual metal contamination when applying the Welch *t*-test to DTW values.

The levels of metals used for treatments were within the range detectable in areas of concern like industrial, mining, or roadway sites. From the metals tested, Pb has the strictest environmental limits based on international guidelines to protect human health (Table 2). Pb is toxic and shows a strong response in this moss species regardless of the experimental settings and analysis methods tested in this study. On the other hand, Zinc could only be detected at a much higher threshold, which may hint that larger doses are needed for plants to be negatively affected. At this time, the treatment levels of Zn tested as part of this work are low enough not to be of concern to public health.

It is important to note that though there may be accumulation of metals in soil or water, their retention is variable, and that does not mean uptake by mosses will occur. Our experiments mimicked atmospheric deposition, but we acknowledge that overland transport can be administered through wet or dry deposition. Values for measured accumulation documented by EPA ambient air quality monitoring programs or via studies of vegetation have been compiled in Table 3.

This study’s lowest detected amounts of metals overlap with the reported environmental values (Table 2). Some of the treatment levels represent a yearly deposition inventory that would match a single dose administered in our experiment. However, previous work has shown that Cu provided in a single dose or cumulative treatments provides similar results in bryophytes [23]. Consideration would then need to be given to a plant, such as moss, with a long enough life span to monitor long-term accumulation and the potential to record inputs from more recent events. Many values in Table 3 (mg/m^2^; mg/L) provide a baseline level for anthropogenic contamination in industrialized, urban areas or near highways. This present study suggests that a mixture of metals is easy to identify, but extra analysis is needed to ensure proper identification of individual metal presence. It is recommended that other metals, metal combinations, and overlaps between metals and other stressors should be further explored to better understand the LIF response recorded in mosses. Other studies have applied transpiration or photosynthetic rate measurements to accompany stress detection, which would help confirm changes identified by LIF corresponding to stress induced via any trial tested [25,33,39]. It would also help to gauge how much stress a particular moss might be under before, during, and after experimentation.

There are several studies looking at the impact of environmental stressors on the health and response of plants. These responses can be highly variable based on the type of vegetation and the environmental factors in a particular ecosystem. Lignified vascular plants often have more complex responses to stress than more simplistic non-lignified plants like mosses. Understanding how each may respond through changes in photosynthetic efficiency could greatly improve the broad application of the LIF technique [58]. Even amongst a single taxonomic grouplike bryophytes, there can be drastic differences in response to temperature and precipitation directly affecting pigment and health [59,60]. Though there are numerous studies looking at the resiliency and benefit of using moss as bioaccumulators and the time of accumulation, there are far fewer that question natural environmental factors that could impact plant health outside of agriculture. The only consistent metrics appear to be based on water availability, which greatly impacts moss more than even the harshest climates [61,62].

There could be room for experiments that lead to a better understanding of the effect such conditions may have on photosynthetic rate and plant capacity for metal accumulations, especially in the case of vegetation that could be used not only as biomonitors but for bioremediation. As noted in the results, there appears to be a possible correlation between environmental stressors and the blue color channel, while the change in photoperiod may be identified using changes in the green color channel. Perhaps the metabolic processes of moss could be further explored as their documentation was not within the scope of this particular research, which focused on heavy metal detection. It is hypothesized that the metal (Cu, Pb) interacts with the chlorophyll to create a shift in the natural fluorescence of the plant. Therefore, a response in the blue color channel is not expected and may be related to a secondary process in the metabolism associated with protein structures or processes associated with growth. There is a possibility that a response in the blue color channel might be indicative of optimal conditions for growth instead of a stress response. This would help to understand the response documented in the moss tray exposed to Zn, and that the metal was preferentially being used by the plant for processes not associated with chlorophyll. More work would certainly need to be done to further document these responses and if they could be observed in similar species.

With respect to metal location within plant tissues, it is possible in future work to explore chlorophyll concentration and potential a/b ratio changes. This could help to inform if shifts in captured fluorescence color in images are due to stress, metals, or both. If Zn is sequestered into lipid or protein structures instead of within chlorophyll itself, then that would directly impact the fluorescence response recorded. Pairing these methods with a more chlorophyll-specific laser system could also help document if changes are specific to different metals and aid in understanding active organic reactions in addition to plant health (i.e., microbial activity) [63,64]. Future work may explore the impact of these stressors, specifically chlorophyll-a and -b, to better understand plant response through LIF. Continued use of the density difference and DTW methods will be key to measuring differences from control samples and monitoring the technique’s effectiveness.

## 5. Conclusions

After testing several types of stressors in mosses and capturing the results via images of LIF, the work has shown that distinctions can be made between heavy metal stress, environmental stressors, and changes in photoperiod. Density difference is best used for single-color analysis with *t*-test verification of deviation from the control. DTW is most effective for two-color analysis and clear separation of metals from other stressors. Three-color analysis using ratios allows us to separate individual metals from each other or distinguish between similar responses between stressor types observed using density difference or DTW. Further exploration of the combined effect of photoperiod with metal stress is interesting. Metal identification at nmol/cm^2^ levels is possible, though Zn and Cu are more difficult to distinguish at 1 nmol/cm^2^ which is within acceptable environmental background levels for those metals. However, Pb and a mixture of metals can be detected at such low thresholds that represent environmentally significant levels compared to Zn and Cu. The detection limits documented in this research are still within the range of safety and health concerns for early detection of poor industry or mining practices. Future work should aim to adapt the technique for field use, multiple types of vegetation, to monitor the environmental health of plants, and to aid in the detection of contaminant spatial distribution.

## Figures and Tables

**Table 1:** Dosing for the 11 moss trays over seven days separated by group. If not specified, the photoperiod is 10 h. All DI, individual and combined metal solutions were 30 mL.

Group	Tray	Day 1	Day 2	Day 3	Day 4	Day 5	Day 6	Day 7
Control		DI	DI	DI	DI	DI	DI	DI
**Metals**	Copper	DI	1 nmol/cm^2^	DI	10 nmol/cm^2^	DI	100 nmol/cm^2^	DI
	Zinc	DI	1 nmol/cm^2^	DI	10 nmol/cm^2^	DI	100 nmol/cm^2^	DI
	Lead	DI	1 nmol/cm^2^	DI	10 nmol/cm^2^	DI	100 nmol/cm^2^	DI
	Mix	DI	1 nmol Cu/cm^2^ 1 nmol Zn/cm^2^ 1 nmol Pb/cm^2^	DI	10 nmol Cu/cm^2^ 10 nmol Zn/cm^2^ 10 nmol Pb/cm^2^	DI	100 nmol Cu/cm^2^ 100 nmol Zn/cm^2^ 100 nmol Pb/cm^2^	DI
**Environmental**	Nutrients	4-3-6 3 mL diluted with 27 mL DI *	DI	DI	DI	DI	DI	DI
	Drought	N/A	N/A	N/A	N/A	N/A	N/A	N/A
	Flood	2xDI	2xDI	2xDI	2xDI	2x DI	2xDI	2xDI
**Photoperiod**	Long	DI 14 h	DI 14 h	DI 14 h	DI 14 h	DI 14 h	DI 14 h	DI 14 h
	Short	DI 6 h	DI 6 h	DI 6 h	DI 6 h	DI 6 h	DI 6 h	DI 6 h
	Dark	DI N/A	DI N/A	DI N/A	DI N/A	DI N/A	DI N/A	DI N/A

* See Appendix B for further details.

**Table 2:** Natural or international guidelines for metal content in soil, water, and air.

	Cu	Zn	Pb
Soil	50–140 mg/kg	10–300 mg/kg	5–30 mg/kg
Water	-	-	10 lg/L 0.001–0.06 mg/L (uncontaminated)
Air	-	-	0.5 lg/m^3^ 4–20 mg/g (dust)
Lowest Level Tested	0.34 mg/m^2^	-	-
Highest Level Tested	34 mg/m^2^	-	-

[10,45,51].

**Table 3:** Examples of measured wet and dry deposition values for Cu, Zn, and Pb.

**Wet Deposition**		
**Cu**	**Zn**	**Pb**
0.49–2.2 mg/m^2^/yr New Jersey [52]	2.41 mg/m^2^/yr Gary, Indiana [53]	1.06 mg/m^2^/yr Gary, Indiana [53]
0.70 mg/m^2^/yr Reston, Virginia [54]	-	2.20 mg/m^2^/yr Chicago, Illinois [55]
1.06 mg/m^2^/yr Chicago, Illinois [54]	-	-
0.8 ± 0.7 mg/m^2^/yr Urban China [56]	-	-
4.7 mg/m^2^/yr Hong Kong, China [56]	-	-
14.6 mg/m^2^/yr Singapore [56]	-	-
**Dry Deposition**		
**Cu**	**Zn**	**Pb**
3.65 mg/m^2^/yr Michigan [57]	-	1.10 mg/m^2^/yr Michigan [57]
21.9 mg/m^2^/yr Chicago, Illinois [57]	-	25.55 mg/m^2^/yr Chicago, Illinois [57]

## Data Availability

The Matlab code can be accessed via https://github.com/KTruax/LIF_image_analysis_moss_stress.git. accessed on 31 July 2023. Code and images analyzed for this study can be found in the Google Drive Folder: Plants—https://drive.google.com/drive/folders/1-54jDySvpvqVb6DrSNZR6gjE_xTPKmiH?usp=sharing. accessed on 31 July 2023.

## Appendix A

*Thuidium plicatile* Mitt. (see also *Thuidium plicatum* Mitt.) is a moss species indigenous to Hawaiʻi [47] and the one chosen to be used for experimentation and observation. The specific specimen used was collected from Oʻahu along the Waʻahila Ridge Trail. A frond-like species in appearance, *Thuidium plicatile* Mitt. is similar in appearance to the invasive *Hypnum plumaeforme* Wilson [65,66] which is not recorded as being present on Oʻahu [47]. *Thuidium plicatile* Mitt., *Thuidium plicatum* Mitt. and *Thuidium plicatum* Mitt. var. *brevifolium* E.B. Bartram is more broadly recognized as a synonym of *Thuidium cymbifolium* Dozy & Molk [66,67]. *Thuidium* is a genus of moss in the family *Thuidiaceae,* so named for the fronds that look like small cedar trees with creeping, branching and pinnate leaves. One hundred ninety-one (191) accepted species names are known in the genus *Thuidium,* all existing in temperate to tropical climates [68]. With that knowledge, if you are encouraged to work with a moss species, you do not have to rely solely upon *Thuidium plicatile* Mitt. as a means of conducting research, as there are many other species in existence.

## Appendix B

Miracle-Gro AeroGarden Liquid Plant Food 4-3-6
Total Nitrogen ……………………………. 4% 1% Ammoniacal Nitrogen 3% Nitrate Nitrogen Available Phosphate (P_2_O_5_) ……………. 3% Soluble Potash (K_2_O) …….…………….. 6% Calcium (Ca) ……………………………. 1% Magnesium (Mg) ……………………….. 0.5% 0.5% Water-Soluble Magnesium
Derived from: Potassium Nitrate, Calcium Nitrate, Mono Potassium Phosphate, Ammonium Nitrate, Magnesium Sulfate
